# Home Sleep Apnea Testing Over 25 Years: A Bibliometric Analysis of Global Research Trends

**DOI:** 10.7759/cureus.117063

**Published:** 2026-09-28

**Authors:** Mélysiane Marcotte, Melissa S Lee, Tanya Chen, Lili HP Nguyen, Joshua Gurberg

**Affiliations:** 1 Department of Medicine, McGill University, Montreal, CAN; 2 Department of Otolaryngology - Head and Neck Surgery, University of Toronto, Toronto, CAN; 3 Department of Otolaryngology - Head and Neck Surgery, McGill University, Montreal, CAN

**Keywords:** early recognition, home sleep apnea testing, obstructive sleep apnea, research trends, technologies

## Abstract

Home sleep apnea tests (HSATs) have emerged as alternatives for diagnosing obstructive sleep apnea (OSA). We conducted a 25-year bibliometric analysis to characterize international research trends in Type 3 and 4 HSATs, focusing on publication, authorship, country of origin, HSAT type, and study design.

MEDLINE, Web of Science, and Embase were searched for studies published from January 2000 to December 2025. Publications primarily evaluating Type 3 or 4 HSATs for OSA diagnosis in children or adults were included. Linear regression and Cochran-Armitage trend tests were used to assess temporal trends.

The literature search yielded 6755 articles, with 487 articles included in the final analysis. From 2020 to 2025, the top five journals of publication were respiratory and sleep-related journals. Publication output increased significantly over time, at a rate of 1.127 articles per year (p < 0.001). From 2020 to 2025, the five most common journals were respiratory or sleep-related. The United States accounted for the largest proportion of publications (33.3%), followed by China and Canada. The proportion of authors with medical degrees increased by 1.08% annually (p = 0.042), while PhD and MD/PhD authorship remained stable. The number of institutions and departments represented among authors also remained stable (p = 0.248 and p = 0.323, respectively).

To the best of our knowledge, this is the first bibliometric analysis of research trends in Type 3 and 4 HSATs. HSAT research has expanded substantially over the past 25 years, with increasing publication output and multi-authorship. Diagnostic cohort studies have consistently dominated the literature, while recent growth in secondary research suggests increasing maturity of the field and capacity for evidence synthesis. Rising MD authorship alongside stable PhD and MD/PhD representation suggests increasing clinician involvement. The United States remains the leading contributor, potentially reflecting greater access to research funding and resources.

## Introduction and background

Obstructive sleep apnea (OSA) is a common respiratory disorder characterized by recurrent apnea events caused by narrowing or collapse of the upper airways [1]. It is an important public health concern due to its high global prevalence, risk of comorbidities, and associated health risks [2]. Despite this, diagnosis is often severely delayed due to limited access to testing and long wait times for specialist referrals. The most common diagnostic methods include polysomnography (PSG) and home sleep apnea testing (HSAT). PSG remains the gold standard for diagnosing OSA due to its validity and accuracy [2]. However, PSG must be performed in an overnight sleep laboratory; therefore, it is not widely accessible due to its high costs and labour-intensive procedures [1]. Thus, HSATs have emerged in recent years as an alternative diagnostic method. They are classified into four categories based on level of supervision, number of channels, and type of channels used [3]. Sleep monitors are classified as Types 1-4, where PSG is a Type 1 device for in-laboratory testing, and Types 2-4 are portable sleep monitors performed at home [4]. According to the American Academy of Sleep Medicine (AASM) criteria, a Type 3 HSAT has four to seven channels, typically including airflow, respiratory effort, heart rate or ECG, and oxygen saturation [4]. Type 4 HSATs have one to two channels, with one of them being oximetry [4].

Over time, HSATs have become more prominent for diagnosing OSA due to their patient-centered approach of simplified home sleep testing in a comfortable setting, lower costs, and easier accessibility compared to PSG [5,6]. As healthcare systems increasingly seek scalable alternatives to laboratory-based PSG, understanding how research on these technologies has evolved is important for identifying where evidence has accumulated and where knowledge gaps remain. With the continued development and advancement of technology, increasing numbers of researchers from highly industrialized regions such as the United States (US), Canada, and the United Kingdom (UK) have devoted themselves to the field of sleep apnea [7]. However, despite the increasing clinical relevance and research activity surrounding Type 3 and Type 4 HSATs, the evolution and distribution of this literature have not been systematically characterized. Bibliometric analysis is an emerging method for assessing research performance. This method quantitatively describes publication patterns on a certain topic, in a given field, institute, or country. In addition, bibliometric analysis can characterize publication output in a specific field and identify temporal, geographic, and thematic trends as well as potential gaps in research activity. Therefore, mapping the Type 3 and Type 4 HSAT literature may provide insight into whether research activity has paralleled their growing role in OSA diagnosis and where further investigation is needed. Accordingly, this bibliometric analysis aimed to characterize trends in Type 3 and Type 4 HSAT research and identify areas warranting further investigation.

## Review

Methods

Study Design and Search Strategy

This study was exempt from institutional review board ethics approval as no patient data was included. A review of the literature was performed through a search of the MEDLINE, Web of Science, and Embase databases from January 1, 2000, to December 31, 2025. The final search was conducted on March 30, 2026. The search strategy was developed in consultation with an experienced librarian, and the complete search strategies for each database are provided in Appendices A-C.

Publications whose primary objective was the evaluation of a Type 3 or 4 HSAT in the diagnosis of OSA were included. The AASM's classification of HSAT was used to determine HSAT level if it was not specified in the article [4]. Exclusion criteria included publication before 2000, non-English publications, studies for which full text was unavailable, studies not evaluating Type 3 or Type 4 HSAT, and studies evaluating outcomes outside the scope of this review (Figure 1).

**Figure 1:**
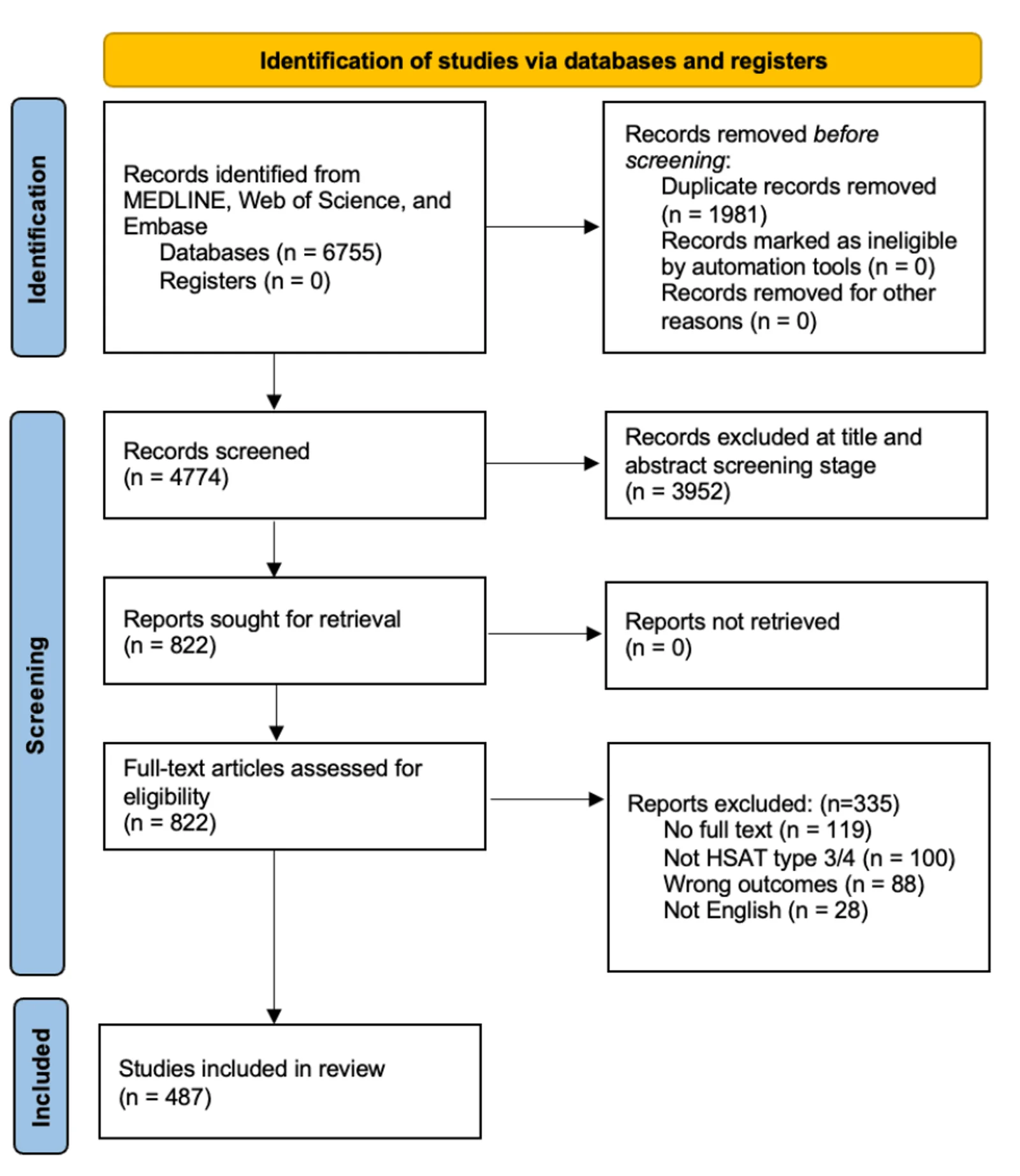
PRISMA flow diagram HSAT: home sleep apnea test

Study Selection and Reviewer Agreement

Prior to screening, the investigators underwent a period of training in article classification. Inter-rater agreement was assessed using Fleiss’ kappa on a sample of 50 journal articles independently classified by each reviewer. Disagreements were discussed among the reviewers and, when necessary, with the senior authors until consensus was reached. Adequate inter-rater agreement was predefined as a Fleiss’ kappa >0.80. Three authors (TC, ML, MM) then divided the remaining articles for title and abstract screening. Most duplicates were automatically identified and removed using Covidence (Veritas Health Innovation, Melbourne, Australia), while remaining duplicates were manually identified and removed by the three authors responsible for screening. Full-text review and data extraction were conducted, divided among the same three authors, with disagreements or uncertainties resolved through discussion and consultation with the senior authors when necessary.

Data Extraction

Overall, 487 articles met the inclusion criteria and were included in the final analysis. Data extracted from each article included year of publication, journal of publication, and impact factor (IF), country of origin, number of institutions, number of departments, number of authors, authors’ educational degrees, and study design. Country of origin was defined according to the first author's institutional affiliation. Institutions and departments were identified based on the affiliations listed for all authors. Authors’ educational degrees were categorized as described in the authors' affiliations or by an online search when not available. Missing data were recorded as missing, and no data imputation was performed. IFs were obtained from the 2025 edition of the Journal Citation Reports (JCR) [8]. Risk-of-bias assessment of individual studies was not performed because the objective of this bibliometric analysis was to characterize publication patterns and research trends rather than to synthesize clinical outcomes or estimate treatment or diagnostic effects.

Statistical Analysis

Statistical analyses were performed using SAS (version 9.2; SAS Institute, Cary, North Carolina). Statistical significance was defined as p < 0.05. Simple linear regression was used to evaluate temporal changes in publication and authorship characteristics, with calendar year as the independent variable and the annual outcome of interest as the dependent variable. Regression coefficients (β) represent the estimated absolute change in each outcome per year and were reported with the 95% confidence interval (95% CI).

Results

Journal of Publication and IF

A total of 487 articles met the inclusion criteria. There was a large variety in journals of publication, resulting in 169 different journals over the study period. Table 1 describes the five most common journals of publication, all of which were respirology- or sleep medicine-based, comprising 34.3% of total articles published between 2000 and 2025.

**Table 1:** Journals with highest publication rates on HSAT Types 3 and 4 from 2000 to 2025 Impact factors were recorded for the year 2025 [8]. HSAT: home sleep apnea test

Rank	Journal title	Article counts	Impact factor
1	Journal of Clinical Sleep Medicine	63	2.7
2	Sleep Medicine	42	3.4
3	Sleep	22	4.9
4	Chest	21	8.6
5	Sleep and Breathing	19	2.0

Origin of Studies

Figure 2 shows trends in publication output by region. The most common country of origin was the US (n = 162, 33.3%), followed by Canada (n = 28, 7.8%) and China (n = 24, 4.9%). The five countries with the most publications outside of these three countries included the UK, Germany, Spain, Italy, and Israel. Globally, publication output increased by an estimated 1.127 articles per year (95% CI, 0.716-1.538; p < 0.001) between 2000 and 2025 (Figure 3). In 2000, three articles were published compared with 30 articles in 2025. Publication output increased significantly over time in the US, China, and other countries, with estimated annual increases of 0.264 articles/year (95% CI, 0.030-0.498; p = 0.028), 0.088 articles/year (95% CI, 0.025-0.151; p = 0.008), and 0.753 articles/year (95% CI, 0.499-1.007; p < 0.001), respectively. Canada demonstrated an estimated annual change of 0.022 articles/year (95% CI, -0.042 to 0.085; p = 0.483).

**Figure 2:**
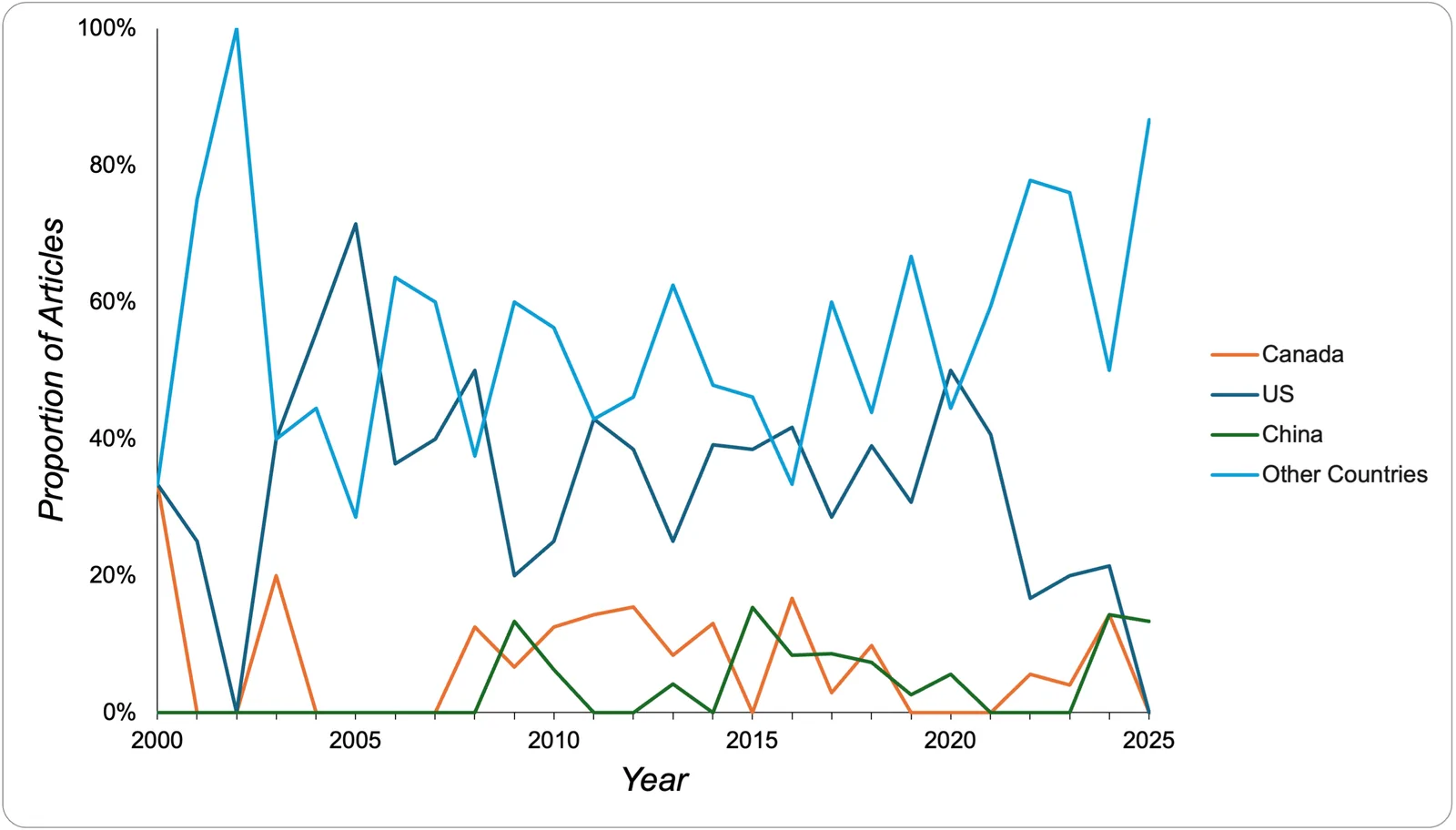
Trends in publication output by region from 2000 to 2025

**Figure 3:**
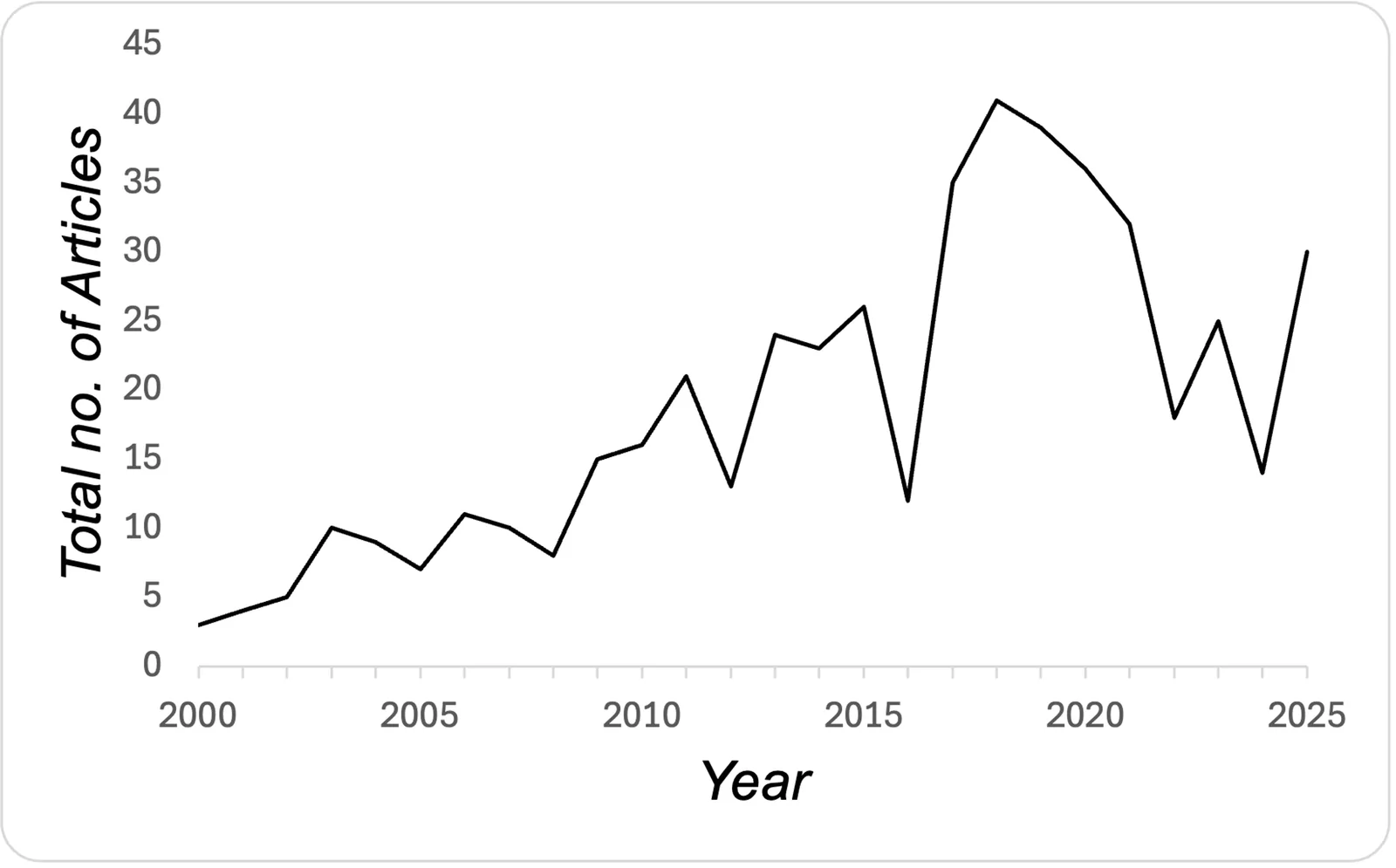
Annual publication output from 2000 to 2025

Authorship

Table 2 shows the overall publication and authorship trends. The average number of authors per article increased from 1.8 in 2000 to 8.1 in 2025, corresponding to an estimated annual increase of 0.120 authors per article per year (p = 0.008). There was also a significant annual increase in the number of authors with a medical degree (MD) (0.108 authors per article per year, p < 0.001). However, the number of authors with a post-doctoral degree (PhD) or a combined MD/PhD degree did not significantly change between 2000 and 2025 (p = 0.338 and p = 0.420, respectively). There was no increase in the number of institutions and departments among authors (p = 0.248 and p = 0.323, respectively).

**Table 2:** Characteristics of studies by year of publication with annual growth rates per year (no./year) from 2000 to 2025 Values are rounded to the nearest whole number. MD: Doctor of Medicine; PhD: Doctor of Philosophy

Year	Total no. of articles	No. of authors per article	Author degrees per article			No. of institutions per article	No. of departments per article
			MD	PhD	MD/PhD		
2000	3	1.8	1.3	1.3	0.7	3.2	2.1
2001	4	3.6	1.2	0.0	0.0	2.6	2.5
2002	5	6.3	1.4	1.4	1.2	2.7	2.7
2003	10	6.8	1.6	0.6	0.5	3.1	2.7
2004	9	6.9	1.4	0.4	0.3	2.7	2.1
2005	7	6.9	2.0	1.4	1.3	3.3	2.7
2006	11	7.1	1.3	0.9	0.7	4.1	3.1
2007	10	2.7	1.4	0.3	0.2	2.0	1.4
2008	8	5.5	1.4	0.5	0.4	2.2	2.0
2009	15	6.1	1.9	1.0	0.7	4.5	1.4
2010	16	4.0	2.5	0.8	0.6	2.5	1.5
2011	21	4.3	2.5	0.9	0.7	2.0	1.9
2012	13	4.6	2.6	1.2	0.9	1.5	2.2
2013	24	4.9	2.5	0.8	0.6	1.9	1.9
2014	23	6.1	3.0	1.3	1.0	2.3	2.2
2015	26	5.5	2.9	0.6	1.1	2.5	2.0
2016	12	6.3	3.1	2.1	0.8	2.6	2.4
2017	35	5.6	3.6	1.2	0.4	3.1	2.1
2018	41	5.8	3.6	0.5	0.7	2.9	2.0
2019	39	5.2	3.8	0.6	0.6	2.4	2.8
2020	36	9.0	4.5	1.1	1.3	4.5	2.5
2021	32	9.0	3.6	0.9	0.6	4.0	4.0
2022	18	8.7	3.9	0.9	1.1	3.8	2.8
2023	25	7.2	2.4	1.4	0.7	3.3	2.7
2024	14	5.6	2.5	0.7	0.4	3.1	2.2
2025	30	8.1	3.6	1.1	0.6	3.6	2.3
Statistics							
Linear regression coefficient, β (no./year)	1.127	0.120	0.108	0.011	0.007	0.024	0.015
95% CI	0.716-1.538	0.035-0.205	0.078-0.137	-0.013-0.035	-0.011-0.025	-0.018-0.067	-0.015-0.045
p-value	<0.001	0.008	<0.001	0.338	0.420	0.248	0.323

Study Type

The distribution of study designs across the 25-year period reflects the progressive maturation of the HSAT field (Figure 4). From 2000 to 2009, the literature consisted exclusively of diagnostic cohort studies, consistent with a field in its early validation phase. Study type diversification began around 2010, with the emergence of feasibility, secondary research, and other clinical study designs. Diagnostic cohort studies remained the dominant study type throughout, peaking proportionally between 2016 and 2021 at 53.3% of all publications. Over the most recent four years (2022-2025), secondary research, including systematic reviews and meta-analyses, accounted for 20.7% of publications, reflecting a field now sufficiently mature to support evidence synthesis. Concurrently, feasibility and engineering studies have grown since 2019, driven by the rapid emergence of novel wearable and artificial intelligence-augmented HSAT technologies.

**Figure 4:**
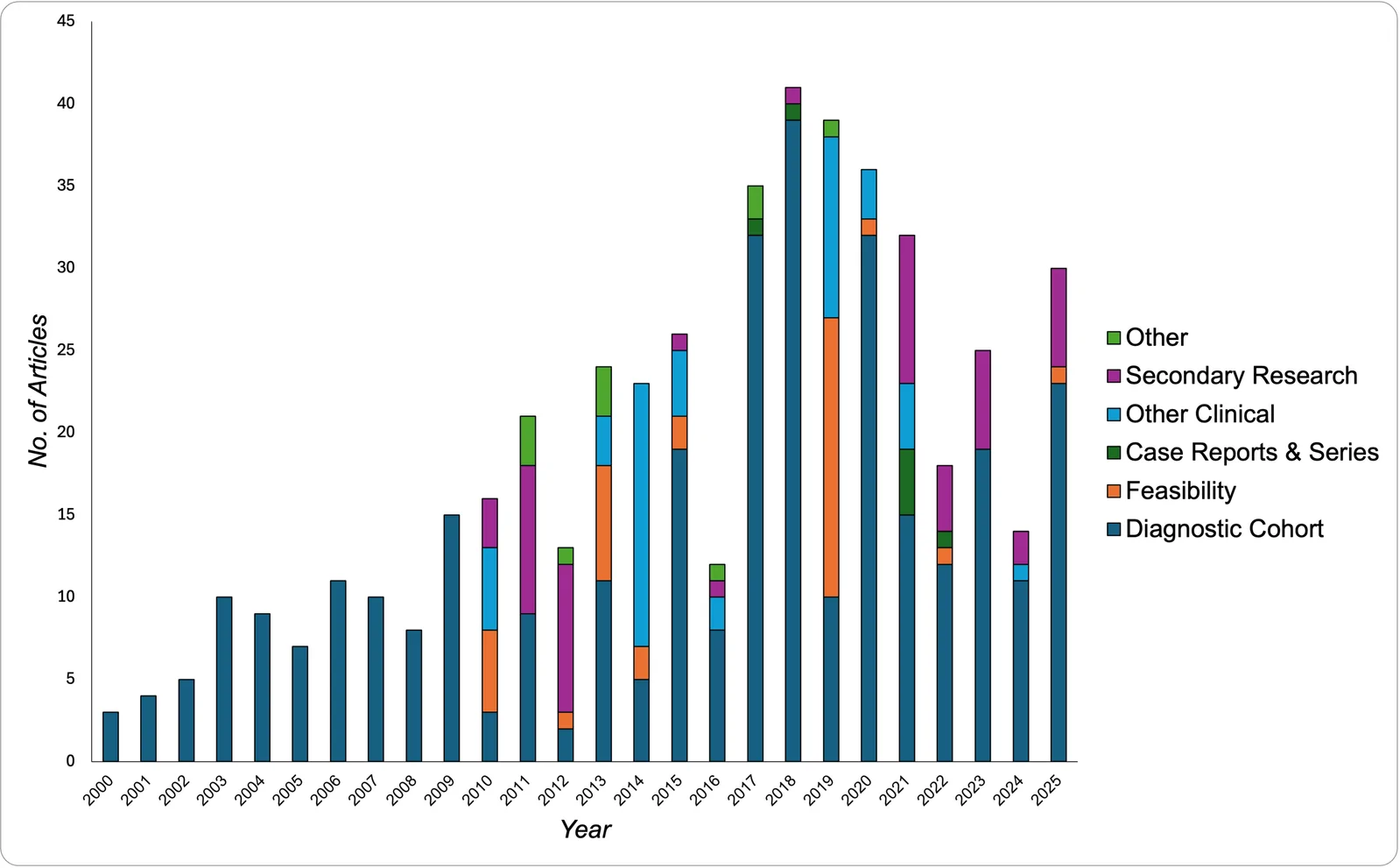
Types of studies published from 2000 to 2025

Discussion

As the prevalence of OSA continues to grow, the importance of its timely diagnosis has concurrently increased in the sleep medicine community, translating to a rapid rise in the number of sleep centers [9] and development of HSAT devices within countries with high quality of life indices [2]. The increasing use and growing integration of HSATs into both academic centers and private practice centers is likely secondary to their widespread availability and accessibility. For clinicians, researchers, and funding agencies alike, understanding the trajectory of this literature is essential to identifying research gaps, avoiding duplication of effort, and directing resources toward the most impactful areas of inquiry.

Our bibliometric analysis demonstrated growing research interest in HSATs, reflected by a significant increase in publication output over the past 25 years. This trend may parallel the expanding availability of OSA screening technologies, including smartwatches, bed sensors, and radiofrequency sensors [10]. Most articles involved collaboration across multiple departments, reflecting the multidisciplinary nature of OSA management [11]. Given the heterogeneity of OSA presentation, pathophysiology, and comorbidities, collaboration among specialties including otolaryngology, respirology, and pediatrics is essential. The increasing emphasis on collaborative research suggests that the field is adapting to this clinical complexity. As OSA and its diagnostic technologies continue to evolve, ongoing evaluation of research trends will remain important.

As technology continues to advance, the demand for HSATs has driven substantial growth in both clinical and engineering research. The distribution of study types suggests that HSAT research has matured over time. Diagnostic cohort studies dominated the early literature, while feasibility studies, clinical trials, and secondary research became increasingly common after 2010. This trajectory mirrors the natural lifecycle of an emerging diagnostic technology, from proof-of-concept to clinical validation to evidence synthesis, which has important implications for how the field should be prioritized going forward. Specifically, the recent growth in systematic reviews and meta-analyses, which accounted for 20.7% of publications between 2022 and 2025, suggests a maturing evidence base for HSATs that may increasingly contribute to clinical guidelines and payer decisions. The growing interest in research and product development in the field of diagnostic sleep medicine is also likely being driven by increased funding from all sources, especially industry funding [12]. Svider et al. (2012) demonstrated that faculty members who received National Institutes of Health (NIH) funding had significantly higher research productivity and impact than non-funded authors [13], and cuts to UK research funding have been associated with reduced research output [14]. These findings underscore the central role of funding in shaping this literature. As technology continues to make significant strides, many start-ups and engineering programs have entered the high-demand HSAT market to launch their products and innovations, resulting in more feasibility and engineering studies [15]. The increase in MD authorship, without a corresponding rise in PhD or MD/PhD authorship, reinforces the interpretation that the field is shifting from basic science toward clinical application, a transition with direct relevance to practicing otolaryngologists and sleep physicians.

The correlation between OSA research productivity and the economic strength of countries is well-known [16], which is demonstrated in our present study with the US as the leading country in HSAT research activity within the global research arena. Our findings are in accordance with a previous bibliometric study, which showed the US produced over 41% of OSA-related publications over a 16-year period, followed by Canada, comprising 7.2% of studies [17]. High-resource countries like the US, Canada, and the UK have greater access to academic and industry funding to dedicate to the field of sleep medicine [18]. The predominance of US-based HSAT research may partly reflect the country’s established sleep research infrastructure, supported by federal research initiatives, professional sleep societies, and collaborative research networks [19]. Although Canada ranked second in total publication output, its annual growth rate was not statistically significant over the study period (0.022 articles/year, p = 0.483). This finding should be interpreted cautiously and may reflect the relatively small number of Canadian publications included in the analysis. Nevertheless, Canada remains an important contributor to HSAT research and has played a significant role in advancing the field. Beyond North America, our study demonstrated that approximately half of publications originated from countries outside the US and Canada, with notable contributions from China, Germany, Spain, Italy, and Israel. Low-resource nations continue to demonstrate low OSA publication activity, likely reflecting the cost and technical complexity of sleep medicine infrastructure and the competing burden of communicable and nutritional diseases [20,21]. China's contribution to HSAT research has increased significantly over the study period (0.088/year, p = 0.008). This finding is consistent with previous studies demonstrating rapid growth in OSA-related research from China [16]. A study by Pan et al. showed that China’s contribution to OSA-related research has grown rapidly in the past decade in comparison to the US, as evidenced by the advancements in technology and commercialization of products in China [7].

Limitations

This study had several limitations. Data were extracted independently by three reviewers, and despite rigorous rater training and high inter-rater reliability (Fleiss Kappa > 0.8), some risk of classification discrepancy remains. Although Medline, EMBASE, and Web of Science are among the most comprehensive biomedical databases, other resources such as Scopus, PsycNet, and CINAHL were not searched and may contain relevant publications. Finally, this analysis does not capture qualitative dimensions of research impact such as citation counts, funding amounts, or clinical guideline adoption, which represent important avenues for future bibliometric work in this field.

Future directions

Future bibliometric work should move beyond publication volume and examine citation impact, research quality, and the influence of HSAT research on clinical practice guidelines. Given the rapid emergence of artificial intelligence-assisted and wearable diagnostic technologies, continued surveillance of publication trends will be important to determine whether these innovations translate into meaningful clinical adoption.

## Conclusions

To the best of our knowledge, this is the first bibliometric analysis to specifically characterize research trends in Type 3 and Type 4 HSATs over a 25-year period. Research in this field has grown substantially, with increasing publication output, rising multi-authorship, and a progressive shift from early-phase validation studies toward clinical application and evidence synthesis. Although Canada ranked second in overall publication output, its growth rate did not significantly increase over the study period. Highly industrialized countries, led by the US, continue to dominate the literature, though China's rapidly growing contribution signals a shifting global landscape. Given the increasing number of secondary research studies published in recent years, the HSAT literature now appears sufficiently mature to support large meta-analyses and may eventually contribute to future clinical guideline development. Artificial intelligence and wearable devices also appear to represent the next frontier driving primary research in the field.

## Appendices

Appendix A

Medline Search Strategy (Searched March 30, 2026)

1. exp Sleep Apnea Syndromes/

2. (sleep* adj3 ((disorder* adj2 breath*) or apnea* or apnoea* or hypopnea* or hypopnoea*)). tw,kf.

3. 1 or 2

4. Monitoring, Ambulatory/

5. Biosensing Techniques/

6. Wearable Electronic Devices/

7. ((home* or portable* or ambulator* or outpatient* or self*) adj5 (test* or monitor* or detect* or diagnos*) adj3 sleep*). tw,kf.

8. or/4-7

9. 3 and 8

Appendix B

Embase Search Strategy (Searched March 30, 2026)

1. exp sleep disordered breathing/

2. (sleep* adj3 ((disorder* adj2 breath*) or apnea* or apnoea* or hypopnea* or hypopnoea*)). mp. [mp = title, abstract, heading word, drug trade name, original title, device manufacturer, drug manufacturer, device trade name, keyword, floating subheading word, candidate term word]

3. 1 or 2

4. ambulatory monitoring/

5. biosensor/

6. ((home* or portable* or ambulator* or outpatient* or self*) adj5 (test* or monitor* or detect* or diagnos*) adj3 sleep*). mp. [mp = title, abstract, heading word, drug trade name, original title, device manufacturer, drug manufacturer, device trade name, keyword, floating subheading word, candidate term word]

7. or/4-6

8. 3 and 7

Appendix C

Web of Science Search Strategy (Searched March 30, 2026)

1. (TS = sleep apnea*) AND LANGUAGE: (English)

2. (TS= (sleep* NEAR/3 (disorder* NEAR/2 breath*) OR apnea*)) AND LANGUAGE: (English)

3. (#2 OR #1) AND LANGUAGE: (English)

4. (TS = ambulator* monitor*) AND LANGUAGE: (English)

5. (TS = ((home* OR portable* OR ambulator* OR outpatient* OR self*) NEAR/5 (test* OR monitor* OR detect* OR diagnos* OR biosens*) NEAR/3 sleep*)) AND LANGUAGE: (English)

6. #5 OR #4

7. #6 AND #3
